# Frequency of KCNC3 DNA Variants as Causes of Spinocerebellar Ataxia 13 (SCA13)

**DOI:** 10.1371/journal.pone.0017811

**Published:** 2011-03-29

**Authors:** Karla P. Figueroa, Michael F. Waters, Vartan Garibyan, Thomas D. Bird, Christopher M. Gomez, Laura P. W. Ranum, Natali A. Minassian, Diane M. Papazian, Stefan M. Pulst

**Affiliations:** 1 Department of Neurology, University of Utah, Salt Lake City, Utah, United States of America; 2 Departments of Neurology and Neuroscience, University of Florida, Gainesville, Florida, United States of America; 3 Department of Neurology, University of Washington, Seattle, Washington, United States of America; 4 Veterans Affairs Medical Center, Seattle, Washington, United States of America; 5 Department of Neurology, University of Chicago, Chicago, Illinois, United States of America; 6 Department of Genetics, Cell Biology and Development and Institute for Translational Neuroscience, University of Minnesota, Minneapolis, Minnesota, United States of America; 7 Department of Physiology, University of California Los Angeles, Los Angeles, California, United States of America; 8 Brain Institute, University of Utah, Salt Lake City, Utah, United States of America; University of Texas M. D. Anderson Cancer Center, United States of America

## Abstract

**Background:**

Gain-of function or dominant-negative mutations in the voltage-gated potassium channel KCNC3 (Kv3.3) were recently identified as a cause of autosomal dominant spinocerebellar ataxia. Our objective was to describe the frequency of mutations associated with *KCNC3* in a large cohort of index patients with sporadic or familial ataxia presenting to three US ataxia clinics at academic medical centers.

**Methodology:**

DNA sequence analysis of the coding region of the *KCNC3* gene was performed in 327 index cases with ataxia. Analysis of channel function was performed by expression of DNA variants in *Xenopus* oocytes.

**Principal Findings:**

Sequence analysis revealed two non-synonymous substitutions in exon 2 and five intronic changes, which were not predicted to alter splicing. We identified another pedigree with the p.Arg423His mutation in the highly conserved S4 domain of this channel. This family had an early-onset of disease and associated seizures in one individual. The second coding change, p.Gly263Asp, subtly altered biophysical properties of the channel, but was unlikely to be disease-associated as it occurred in an individual with an expansion of the CAG repeat in the *CACNA1A* calcium channel.

**Conclusions:**

Mutations in KCNC3 are a rare cause of spinocerebellar ataxia with a frequency of less than 1%. The p.Arg423His mutation is recurrent in different populations and associated with early onset. In contrast to previous p.Arg423His mutation carriers, we now observed seizures and mild mental retardation in one individual. This study confirms the wide phenotypic spectrum in SCA13.

## Introduction

Dominant spinocerebellar ataxias (SCA) are heterogeneous neurological diseases with phenotypes consisting of cerebellar ataxia, extrapyramidal signs, dysarthria, oculomotor abnormalities, motor neuron signs, cognitive decline, epilepsy, autonomic dysfunction, sensory deficits, and psychiatric manifestations. Currently, twenty-nine distinct SCA loci are known and mutations identified for 17 of the SCAs.

The most common mutation observed is a DNA CAG repeat expansion resulting in an enlarged poly-glutamine domain in the respective protein. In addition to translated CAG-repeat mutations, a number of non-CAG ataxia genes have been identified with functions as diverse as protein kinases, cytoskeletal and mitochondrial proteins, and proteins regulating intracellular calcium stores [1], [2], [3], [4], [5]. We previously described *KCNC3* (Kv3.3, OMIM# 605259 RefSeq NG_008134.1, NP_004968.2 and NM_004977.2 in GenBank) as the gene mutated in SCA13 [6], [7], [8], [9], [10]. Affected individuals in a French pedigree with early-onset ataxia segregated a p.Phe448Leu mutation. A p.Arg420His mutation segregated in a Filipino pedigree with late onset and slow progression of ataxic symptoms. In addition, two families of European descent with early onset ataxia segregated a p.Arg423His mutation.

We now describe the analysis of a large number of ataxia patients from mixed-ethnicity American pedigrees with autosomal dominant or sporadic ataxia of early or late onset. We show that the p.Arg423His mutation, originally described in Europeans, is recurrent in this American cohort.

## Materials and Methods

### Ethics Statement

All subjects gave written consent and all work was approved at their respective institutions: Institutional Review Board at the University of Minnesota; Institutional Review Board at the University of Chicago and the University of Washington Human Subjects Review Committee.

### Identification of Index cases

The study group was composed of 327 index cases from sporadic ataxia patients and from pedigrees with autosomal dominant ataxia. Three 1^st^ degree relatives of index cases with non-synonymous DNA changes were studied in addition. The ethnic composition of these index cases was composed of 61.8% Caucasian, 1.5% African American, 1.2% Hispanic and 35.5% other. In 22.1% of index cases individuals came from pedigrees with autosomal dominant inheritance, in 21.2% ataxia was familial without a clear mendelian pattern. In 49.7% ataxia was sporadic and in 7% of index cases family history was unknown. Index cases had both late as well as early disease onset.

All patients presented to three ataxia subspecialty clinics in academic medical centers and had undergone clinical examinations and genetic tests to exclude ATNX1, ATXN2, ATXN3, CACNA1A, ATNX7, and TBP (OMIM# 164400, 183090, 109150, 183086, 164500, 607136) CAG expansion mutations, although not all gene tests were conducted in all patients.

The control group included 284 alleles contributed by 77 normal individuals living in France, 10 index individuals from French families in the CEPH pedigree set and 55 US individuals that represented spouses of patients with Parkinson disease that were sequenced in a prior study [11]. DNA samples were collected and stored using standard procedures.

Mutation screening and analysis of channel function was done as described in Figueroa et al. 2010 [11] . Each fragment containing mutations was amplified by PCR and sequenced a second time to confirm that the identified mutations were not due to PCR artifacts.

### Electrophysiology

Mutations were made in a human wild-type Kv3.3 cDNA clone (kind gift of Dr. James L. Rae) using the QuikChange kit (Strategene) [12]. Mutations were verified by sequencing. Run off transcripts were prepared using the mMessage mMachine kit (Ambion). Wild type and mutant subunits were separately expressed by injecting RNA into *Xenopus* oocytes. Channel activity was recorded 2–3 days later using a two electrode voltage clamp [13]. During electrophysiological experiments, oocytes were bathed in a solution containing 4 mM KCl, 85 mM NaCl, 1.8 mM CaCl_2_, and 10 mM HEPES, pH 7.2. To measure tail currents, the bath solution was switched to 89 mM RbCl, 2.4 mM NaHCO_3_, 0.82 mM Ca(NO_3_)_2_, 0.41 mM CaCl_2_ and 10 mM HEPES, pH 7.2. Electrode resistances ranged from 0.3 to 0.8 MΩ.

The voltage dependence of activation was determined from normalized isochronal tail current amplitudes. Wild-type Kv3.3 or p.Gly263Asp currents were evoked by stepping from −90 mV to various test potentials, followed by repolarization to −90 mV. Isochronal tail current amplitudes were normalized to the maximal value obtained in the experiment and plotted versus test potential. Data were fitted with single Boltzmann functions to determine the midpoint of activation and slope factor, which are provided as mean ± SEM. These parameters did not differ significantly between wild type and p.Gly263Asp channels (Student's *t*-test). To obtain values for the activation and deactivation time constants, τ_act_ and τ_deact_, currents were fitted with a single exponential function. Values are provided as mean ± SEM. Statistical significance was evaluated using one-way ANOVA (p<0.05).

## Results

### Genetics

Exons 1–4 of the KCNC3 gene were sequenced including exon-intron boundaries for introns 1 and 2 as well as the entire small intron 3. Table 1 summarizes DNA sequence changes found in 327 index cases and 142 controls. All variants were present in the heterozygous state. Exon 5 was not analyzed as it does not contain any coding sequences. We identified a total of 12 allelic variants. These consisted of 11 single nucleotide changes, and one 1 bp duplication. Four variants were located in the coding region. Of these, two resulted in amino acid changes, one changing an arginine to a histidine (p.Arg423His) and one changing a glycine to aspartic acid (p.Gly263Asp). The two remaining coding variants resulted in synonymous changes. Variant c.1257C>A was a 3^rd^ base wobble at amino acid position Val 419 in two related individuals, the other c.1404C>T was also a 3^rd^ base wobble found at amino acid Tyr 468. Although these variants were only found in cases, they likely represent very rare benign polymorphisms as they do not change the amino acid sequence or introduce cryptic splice sites.

**Table 1:** Sequence Variants Found in Index Cases.

Location	Type	Flanking Sequence	Genomic Position	Coding Position	Amino Acid Change	% Cases	% Controls
5′ UTR	Non-coding	CGTCTT**T/G**AAATAG	g.4870T>G			0.60	0.00
5′ UTR	Non-coding	AGCCAA**T/A**CGCTCC	g.5065T>A	c.-231T>A	p. =	0.30	0.00
5′ UTR	Non-coding	CTCCCC**/CC/**TAAGCC	g.5186_5187dup	c.-110_-109dup	p. =	0.15	0.00
5′ UTR	Non-coding	GCCCCC**G/T**CTACCT	g.5203G>T	c.-94G>T	p. =	0.15	0.00
Exon 1	Coding	CGGGCG**G/A**CGCGGG	g.6083G>A	c.788G>A	p.Gly263Asp	0.15	0.00
Exon 2	Coding	CTTCGT**C/A**CGCATC *	g.10682C>A	c.1257C>A	p. =	0.30	0.00
Exon 2	Coding	TTCTGC**G/A**CATCTT	g.10693G>A	c.1268G>A	p.Arg423His	0.30	0.00
Exon 2	Coding	TTACTA**C/A**GCTGAG	g.10829C>A	c.1404C>A	p. =	0.30	0.00
Intron 3	Non-coding	GCCCCC**C/A**CTACTG	g.13799C>A	c.2170+14C>A		0.76	0.70
Intron 3	Non-coding	AGAGGG**G/A**GATGGG	g.13858G>A	c.2170+73G>A		0.46	0.35
Intron 3	Non-coding	CCCAAC**T/C**CTCTGG **	g.14003T>C	c.2171-26T>C		0.15	0.35
Intron 3	Non-coding	GGGGGA**G/A**GAGGCG	g.13868G/A	c.2170+83G>A		0.15	0.00

RefSeq NG_008134.1, NM_004977.2, NP_004968.2, % reflects allele frequency.

*Variant Found in patient with p.Arg423His and sister.

**Known SNP rs35578310.

A number of variants that did not change the predicted amino acid sequence were identified either in the 5′-UTR or in intron 3; none of the 5′-UTR variants were found in the controls. Other than exon-intron boundaries, we did not attempt to sequence introns with the exception of intron 3 owing to its small size. Four sequence variants were detected in this intron. One variant occurred at a slightly higher frequency in cases versus controls, g.13858G>A with an allele frequency 0.46% frequency in cases and 0.35% in controls. This finding is similar to that reported in our previous study, where g.13858G>A in intron 3 was relatively common with an allele frequency of 3.65% in cases. When combining cases from both studies, this variant has a combined total allelic frequency of 0.62% in affected individuals versus 0.35% in controls.

### Phenotypes of Patients with the p.Arg423His mutation

**Figure 1:**
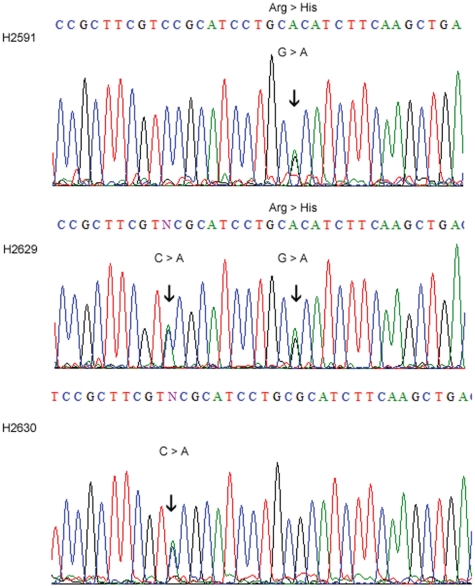
DNA electropherograms of a family with the p.Arg423His mutation. H2591shows the p.Arg423His mutation (arrow, top), her son (H2629) shows the p.Arg423His mutation and the c.1257C>A variant, and her daughter (H2630) shows the c.1257C>A change, but lacks the mutation.

**Figure 2:**
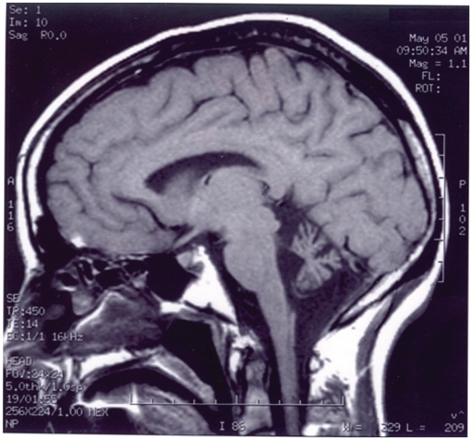
MRI of Patient with the p.Arg423His mutation. Cerebellar atrophy in a 48 year old female (H2591) with ataxia and the KCNC3^Arg423His^ mutation. Midsagittal T1-weighted MRI of the brain shows a small atrophic cerebellum with a normal appearing brainstem.

DNA electropherograms of the family found to carry the p.Arg423His mutation are shown in Figure 1 and their clinical phenotypes in Table 2. The index case (H2591) is a Caucasian female who had onset of the disease in infancy with delayed motor milestones and ataxic gait. At age 48, she presented at the neurology clinic with dysarthria, ataxia, mild horizontal nystagmus, hyperactive tendon reflexes and a normal sensory exam. The patient indicated that she had been clumsy all her life and started walking at 14 months of age. She did not exhibit cognitive impairment with an IQ in the normal range. MR Imaging showed cerebellar midline and cortical atrophy (Fig. 2), EMG and nerve conduction velocities were normal. This patient has been followed for 10 years and has had very little change but there has been some moderate deterioration in her gait. The son (H2629) of the index case had normal initial milestones but was also noted to be delayed throughout childhood with awkward fine motor control and was unable to walk independently until 8 years of age. He developed seizures in infancy, and imaging at age 3 showed a small cerebellum. On examination at age 17, he presented with dysmetria, dysarthria, nystagmus, and hyperactive tendon reflexes. Cognitive impairment was evident with an estimated IQ of 65. Imaging results for the son were not available for publication.

**Table 2:** Clinical and mutation findings of p.Arg423His index patient and relatives.

ID	Index Patient	Age at Onset	Age at Exam	Sign at Onset	Cerebellar Signs	Neurologic Examination	IQ	Imaging	Progression	Coding Position	Amino acid Change
H2591	Yes	Infancy	48	Ataxic gait	Yes	Dysarthria, mild horizontal nystagmus, hyperactive tendon reflexes. Normal sensory exam.	IQ in normal range	Cerebellar Atrophy, EMG/NCV normal	Clumsy all her life walked at age 14 months.	c.1268G>A	p.Arg423His
H2629	No, son of index	Infancy	17	Ataxic gait and seizures	Yes	Dysmetria, dysarthria, nystagmus, hyperactive tendon reflexes.	IQ estimated at 65	Small cerebellum at age 3	All milestones were delayed. Unable to walk independently until 8 years of age.	c.1268G>A	p.Arg423His
H2630	No, daughter of index	NA	15			Normal	Not done				

Nucleotide numbering for cDNA –based nomenclature uses 1+ as to the A of the ATG translation initiation codon in Genebank RefSeq NM_004977.2.

The initiation codon is codon 1. RefSeq NG_008134.1, NP_004968.2.

The daughter (H2630) who did not carry the p.Arg423His mutation was examined at age 15 and had no abnormal neurological findings. The affected son and unaffected daughter both carried a c.1257C>A variant, a 3^rd^ base wobble at amino acid position Val 419 (Fig. 1). It remains possible that this variant may worsen symptoms in carriers of the p.Arg423His mutation.

### Functional Analysis

To characterize the effects of the novel mutation on channel function, mutant p.Gly263Asp was introduced into wild type human Kv3.3 [12]. Wild type and mutant subunits were separately expressed in *Xenopus* oocytes and channel activity was recorded with a two electrode voltage clamp [13].

**Figure 3:**
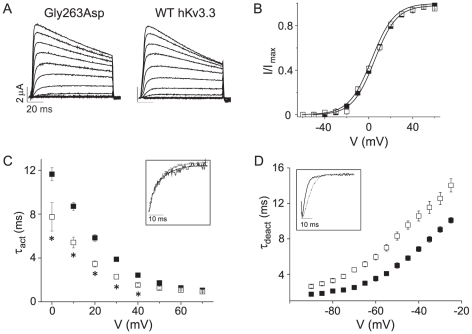
Mutation kinetics in *X. laevis* oocytes. p.Gly263Asp alters activation and deactivation kinetics in *X. laevis* oocytes. (**A**) p.Gly263Asp currents were evoked by stepping from −90 mV to voltages ranging from −90 mV to +70 mV in 10 mV increments. (**B**) Normalized isochronal tail current amplitudes have been plotted versus voltage for wild-type (▪, *n* = 84) and p.Gly263Asp (□, *n* = 8). (**C**) Activation time constant (τ_act_) was plotted versus voltage. *Wild-type (▪, *n* = 36) and p.Gly263Asp (□, *n* = 8) values differed significantly , p<0.05. Inset: Wild-type (solid) and p.Gly263Asp (dotted) currents were evoked at 0 mV, scaled and overlaid. (**D**) Deactivation time constant (τ_deact_) was plotted versus repolarization voltage. Wild-type (▪, *n* = 10) and p.Gly263Asp (□, *n* = 8) values differed significantly, p<0.05. Inset: Wild-type (solid) and p.Gly263Asp (dotted) tail currents were recorded at −60 mV, scaled and overlaid.

The p.Gly263Asp subunit produced functional channels when expressed alone (Fig. 3A). The voltage dependence of activation was determined as a function of voltage and compared to wild type (Fig. 3B). The data sets were fitted with single Boltzmann functions, which yielded half activation voltages of 5.3±0.5 mV and 2.7±0.8 mV and slope factors of 10±0.3 and 10.3±0.7 for wild-type and p.Gly263Asp channels, respectively (Fig. 3B). These values did not differ significantly, indicating that the p.Gly263Asp mutation had no detectable effect on the steady state properties of activation. In contrast, the p.Gly263Asp mutation did alter activation and deactivation kinetics. Evoked currents and tail currents were fitted with single exponential functions to characterize activation and deactivation kinetics, respectively (Fig. 3C, 3D). p.Gly263Asp channels activated more quickly and deactivated more slowly relative to wild type. At 0 mV, p.Gly263Asp channels opened ∼1.5-fold more rapidly than wild type channels (Fig. 3C). At −70 mV, p.Gly263Asp channels closed ∼1.5-fold more slowly than wild type channels (Fig. 3D). Although relatively modest in size, these changes in kinetics were statistically significant.

As the functional changes were significant, but much less severe than the p.Phe448Leu mutation previously observed in a French pedigree, we pursued two additional lines of investigation to determine whether p.Gly263Asp was a rare variant or disease-causing. First, we examined the conservation of the changed amino acid in other species and in other ion channels. The glycine at position 263 is highly variable and not conserved even among Kv3 family members. Second, we investigated whether this patient had any other known mutations associated with a progressive ataxia. Indeed, subsequent analysis revealed a DNA CAG repeat expansion in the CACNA1a gene causing SCA6. The patient carried a pathologic expansion with 23 CAG repeats with a typical pure cerebellar phenotype with onset in midlife. Thus, the KCNC3^Gly263Asp^ allele likely represents a rare benign polymorphism.

## Discussion

We report findings from DNA sequence analysis of the human *KCNC3* gene in a large mixed ethnicity American cohort of autosomal dominant early and late onset ataxia patients. Overall, DNA variants located in exons and exon-intron boundaries were rare. We identified twelve nucleotide changes in 13 sporadic, 5 familial and 2 cases with unknown family history.

Of these, only one variant can be considered disease-causing. The arginine to histidine amino acid change at codon 423 has been observed in two other pedigrees of European ancestry [11]. Similar to those patients, the two individuals carrying the KCNC3^Arg423His^ allele had early-onset and slow progression. One individual had mild mental retardation and seizures as well. Seizures have been previously observed with the KCNC3^Arg420His^ mutation, but not with the KCNC3^Arg423His^ allele. Mental retardation had only been observed with the KCNC3^Phe448Leu^ mutation [11]. Thus, this study confirms the wide phenotypic spectrum associated with KCNC3 mutations.

The p.Arg423His mutation is located in the highly conserved S4 domain of the channel. The S4 domain is thought to act as the main voltage sensing element [14], [15] and is involved in channel activation [16]. Both the p.Arg420His and p.Arg423His mutations exhibited a strong dominant negative effect when expressed at a 1∶1 ratio of wild type to mutant subunit, which is the expected ratio found in SCA13 patients.

We identified another non-synonymous variant that was not present in controls. This novel heterozygous variant c.788G>A, p.Gly263Asp was found in one patient. Expression of this variant in *Xenopus* oocytes showed alterations in activation and deactivations kinetics, but the changes were relatively mild compared with the previously reported KCNC3^Phe448Leu^ mutation that displayed a much more significant gain-of-function in our heterologous expression system [6]. In addition, the glycine at position 263 is conserved in only 44 out of the 164 of the voltage-gated K channel family. Further genetic analysis revealed that the patient carried a repeat-expansion mutation in the CACNA1a gene. His brother, also affected with SCA6, was not found to have the KCNC3 amino acid change. Thus, it is unlikely that the subtle changes in channel function seen *in vitro*, are responsible for ataxia *in vivo*.

As sequencing of the *KCNC3* gene is available in academic and commercial laboratories, it is likely that additional rare DNA variants will be identified. Great caution will need to be exercised in the interpretation of these variants. As the KCNC3^Gly263Asp^ variant shows, even the presence of subtly changed channel function in heterologous cell systems does not prove that a DNA variant is causative *in vivo*. And unless very large numbers of control alleles are examined, it will be difficult to distinguish between disease-associated variants and very rare polymorphisms on purely genetic grounds.

Problems in interpreting our KCNC3^Gly263Asp^ variant are reminiscent of similar issues in a variant of the SCNA (Na_v_1.4) sodium channel subunit. Although a SCNA^Ser906Thr^ variant segregated perfectly with periodic paralysis in several large pedigrees and was shown to alter entry into and recovery from slow channel inactivation, it was later identified in up to 5% of the normal population without apparent association with disease [17].

Our present data support the notion that SCA13 is a rare ataxia and that KCNC3 mutations causing SCA13 have arisen in a number of ethnic groups. Despite the enormous progress that has been made in the understanding of the genetic basis of hereditary ataxia, the origin of many sporadic ataxias remains obscure. In our study, about half of the cohort had sporadic ataxia. Although 62% of the DNA changes were found in these patients, none were disease- causing and found in non-coding regions. A number of previous studies have also looked for gene mutations in sporadic ataxias and the frequency of positive genetic tests in apparently sporadic ataxia patients ranged from 2% to 22% [18], [19], [20], [21]. The discrepancies between studies are probably due to variable genetic background and to different ascertainment of the study population.

Two of the mutations involving arginines in the S4 voltage sensor domain are recurrent in different populations with overall similar phenotypes for the specific mutation. The p.Phe448Leu mutation, although non-recurrent at this point, segregated in a large pedigree and resulted in significantly altered biophysical properties of the KCNC3 channel. As demonstrated by the KCNC3^Gly263Asp^ variant, great care needs to be exercised when interpreting other rare coding changes even when they show subtle changes in a heterologous expression system.
